# Evaluation of Central Venous Catheter Associated Blood Stream Infections: A Microbiological Observational Study

**DOI:** 10.1155/2013/936864

**Published:** 2013-07-09

**Authors:** Vinay Khanna, Chiranjay Mukhopadhayay, Vandana K. E., Murlidhar Verma, Partha Dabke

**Affiliations:** ^1^Department of Microbiology, Kasturba Medical College, Manipal University, Karnataka 576104, India; ^2^Department of Medicine, Kasturba Medical College, Manipal University, Karnataka 576104, India; ^3^Kasturba Medical College, Manipal University, Karnataka 576104, India

## Abstract

There are substantial morbidity and mortality associated with vascular catheter use among crictically ill patients. The attributable mortality is 10% to 25% which is associated with bacteremia among those who are hospitalized. This study was undertaken to identify catheter related blood stream infections, to isolate pathogenic microorganisms present in intravascular catheter related local infections, exit site infections, and to determine the predisposing factors for the development of such infections and antibiotic sensitivity pattern of the isolated organisms in tertiary care hospital.

## 1. Introduction

Intravascular catheters are indispensable in modern-day medical practice, particularly in intensive care units (ICUs). Although such catheters provide necessary vascular access, their use puts patients at risk for local and systemic infectious complications, including local site infection, catheter related blood stream infections (CRBSIs), septic thrombophlebitis, endocarditis, and other metastatic infections (e.g., lung abscess, brain abscess, osteomyelitis, and endophthalmitis).

The incidence of CRBSI varies considerably by type of catheter, frequency of catheter manipulation, and patient-related factors (e.g., underlying disease and acuity of illness). Peripheral venous catheters are the devices most frequently used for vascular access. Although the incidence of local or bloodstream infections (BSIs) associated with peripheral venous catheters is usually low, serious infectious complications produce considerable annual morbidity because of the frequency with which such catheters are used. However, the majority of serious catheter-related infections are associated with central venous catheters (CVCs), especially those that are placed in patients in ICUs. In the ICU setting, the incidence of infection is often higher than in the less acute in-patient or ambulatory setting. In the ICU, central venous access might be needed for extended periods of time; patients can be colonized with hospital-acquired organisms; and the catheter can be manipulated multiple times per day for the administration of fluids, drugs, and blood products. Moreover, some catheters can be inserted in urgent situations, during which optimal attention to aseptic technique might not be feasible. Certain catheters (e.g., pulmonary artery catheters and peripheral arterial catheters) can be accessed multiple times per day for hemodynamic measurements or to obtain samples for laboratory analysis, augmenting the potential for contamination and subsequent clinical infection.

Bloodstream infections may be either primary or secondary. Secondary infections are related to infections at other sites, such as the urinary tract, lung, postoperative wounds, and skin. Primary BSIs comprise the majority (64%) of nosocomial BSIs reported to the CDC's National Nosocomial Infection Surveillance (NNIS) system, and most are due to infected intravascular, mostly central venous catheters. The remaining patients with primary BSIs have bacteremia with no identifiable source. More than 250,000 vascular catheter-related bacteremia and fungemia occur annually in the USA with an attributable mortality ranging from 12% to 25% in critically ill patients [1, 2].

### 1.1. Defining Criteria for Catheter Related Blood Stream Infections (CRBSI) and Catheter Associated Blood Stream Infections (CABSI)

*CRBSI.* Bacteremia/fungemia in a patient with an intravascular catheter with at least one positive blood culture obtained from a peripheral vein, clinical manifestations of infections (i.e., fever, chills, and/or hypotension), and no apparent source for the BSI except the catheter. One of the following should be present: a positive semiquantitative (>15 CFU/catheter segment) or quantitative (>102 CFU/catheter segment catheter) culture whereby the same organism (species and antibiogram) is isolated from the catheter segment and peripheral blood.

*CABSI*. Vascular access device terminates at or close to the heart or one of the great vessels. An umbilical artery or vein catheter is considered a central line. BSI is considered to be associated with a central line if the line was in use during the 48-hour period before development of the BSI. If the time interval between onset of infection and device use is >48 hours, there should be compelling evidence that the infection is related to the central line.

## 2. Material and Methods

It was case-control study done in tertiary care hospital. The number of participants in this study was 50 cases and 50 controls. Patients aged >18 yrs, admitted in intensive care units with intravascular catheters, were included. Patients with fever prior to catheter placement and proven source for sepsis other than the intravascular catheter of interest were excluded from this study. The following definitions were used to describe cases and controls.

*Case*. Any patient with proven localized catheter colonization, exit site infection, or bloodstream infection (as per CDC guidelines).

*Control*. Any patient in whom intravascular catheter grows no organism on semiquantitative cultures.

### 2.1. Method of Collection of the Catheter Specimen [3]

Skin was cleaned with 70% alcohol prior to catheter removal. The proximal end of catheter was held and carefully removed from the patient with a sterile instrument, taking care to avoid contact with the skin. The distal end was held over a sterile tube, and tip was cut with a sterile scissors and the terminal 2 to 3 inches dropped into the tube. Drying was avoided by sealing the tube and transporting the same to the laboratory as soon as possible. Catheter tip processing was done by Maki's Roll over plate method and catheter flush culture [4, 5]. In blood processing, blood was collected within 48 hours of catheter collection under all aseptic precautions in a BacT bottle and analyzed using the BacT ALERT system. 

#### 2.1.1. Antibiotic Sensitivity Tests

Antibiotic sensitivity pattern was done by Kirby-Bauer disk diffusion method as recommended by Clinical Laboratory Standard Institute (CLSI). Screening method for the detection of MDR (MRSA, ESBL, and so forth) organism was done by using Oxacillin (1 *μ*g) disk in Mueller Hinton agar plate for MRSA detection and double disc approximation or double disk synergy using Amoxicillin-Clavulanic acid (20/10 *μ*g) and Ceftriaxone (30 *μ*g) at a distance of 30 mm between centers of the two disks. American type culture collections (ATCC) were used as control strains. Statistical analyses were done using SPSS 16.0 for windows and strength of association is expressed as odds ratio which was derived using logistic regression analysis.

## 3. Results

The commonest premorbidity among the controls and patients with CRBSI was renal failure (36% versus 36.4%) while that among the patients with local catheter infections was diabetes (28.2%) and the commonest purpose of cannulation was for administration of IV fluids and parenteral antibiotics as shown in Table 1. Local signs of inflammation like erythema, warmth, induration, tenderness, and purulence at exit site were seen among all patients with CRBSI and among majority of patients with local catheter infections (97.4%).

### 3.1. Type of Organisms

86.12% of the isolates were bacteria, while 11.11% of the pathogens were *Candida* species and only 2.77% were polymicrobial.

### 3.2. Bacteriological Profile of the Cases

64% of the pathogens of CRBSI were Gram positive and 36% were Gram negative while 57.84% of the pathogens causing CRLI were due to Gram-negative organisms and 42.16% were Gram-positive.

### 3.3. Distribution of Pathogens amongst CRBSI

The commonest pathogen of CRBSI was *Staphylococcus aureus *(40%) and the least common was* Acinetobacter baumannii* (4%). *Candida* spp. and *Pseudomonas aeruginosa* both accounted for about 16% of the infections while about 8% infections were caused each by *E. coli*, *K. pneumonia*, and coagulase-negative *Staphylococcus* spp.

### 3.4. Distribution of Pathogens amongst CRLI

The commonest pathogen of CRLI was CONS (19.28%) and the least common were polymicrobials (3.61%). Both *Pseudomonas* and *Acinetobacter* showed growth in 16.87% of the cases followed by *Staphylococcus aureus* (13.25%) and *E. coli* (10.84%). *Klebsiella pneumoniae* and *Candida* spp. each showed growth in 9.64% cases.

### 3.5. Antibiotic Sensitivity Pattern in Catheter Related Local Infection

#### 3.5.1. Gram Positive Bacteria (*Staphylococcus* spp.)

All resistant *Staphylococcus* (MRSA and MRCONS) isolated from local catheter infections were 100% sensitive to vancomycin, teicoplanin, and linezolid and all sensitive *Staphylococcus* (MSSA, MSCONS) were 100% resistant to ciprofloxacin.

#### 3.5.2. Gram Negative Bacteria (Oxidase Positive)

The most sensitive routine antibiotic for *Pseudomonas aeruginosa* strains isolated in CRLI was piperacillin (85.7% sensitive) and the most sensitive reserved antibiotics were cefoperazone-salbactum, piperacillin-tazobactum, ticarcillin-clavulanic acid (85.7% each), and meropenem (78.6%).

#### 3.5.3. Gram Negative Bacteria (Oxidase Negative)

The most sensitive routine antibiotic for *E. coli* isolated in CRLI was cefuroxime (88.9% sensitive) and the most sensitive reserved antibiotic was meropenem (88.9% sensitive) while the most sensitive routine antibiotic for *K. pneumoniae* isolated in CRLI was amikacin (12.5% sensitive) and the most sensitive reserved antibiotic was meropenem (50% sensitive). In case of *A. baumannii *isolated in CRLI, the most sensitive routine antibiotic used was amikacin (35.7% sensitive) and cefoperazone-salbactum in reserved antibiotic (35.7% sensitive).

### 3.6. Antibiotic Sensitivity Pattern in CRBSI

#### 3.6.1. Gram Positive Bacteria (*Staphylococcus* spp.)

All resistant *Staphylococcus* (MRSA) isolated from CRBSI were 100% sensitive to cotrimoxazole and chloramphenicol among routine antibiotic and 100% sensitive to vancomycin, teicoplanin, and linezolid among reserved antibiotics. All sensitive *Staphylococcus* (MSSA and MSCONS) were 100% resistant to ciprofloxacin.

#### 3.6.2. Gram Negative Bacteria (Oxidase Positive)

The most sensitive routine antibiotics for *P. aeruginosa* isolated from CRBSI were piperacillin and ciprofloxacin (100% sensitive each). Among the reserved antibiotics the most sensitive were cefepime, cefoperazone-salbactum, piperacillin-tazobactum, ticarcillin-clavulanic acid, and meropenem (100% sensitive each).

#### 3.6.3. Gram Negative Bacteria (Oxidase Negative)

There were no routine antibiotics sensitive for *E. coli* isolated from CRBSI and both were ESBL producers and among reserved antibiotics meropenem was most sensitive (100%). For *K. pneumoniae *isolated from CRBSI, the most sensitive routine antibiotics were gentamicin, netilmicin, amikacin (all 100% sensitive), and meropenem among reserved antibiotics (100% sensitive). Only one strain of *A. baumannii* isolated from CRBSI was resistant to all routine and reserved drugs (multidrug resistant). As shown in Tables 2 and 3, the incidence of catheter-related infections was the highest among patients with a femoral venous line (36%). This was followed by patients with subclavian, basilic and jugular venous accesses (20% versus 22% versus 22%) as compared to controls. In subjects with triple lumen catheters, 40% cases had catheter related infections as compared with 26% of controls whereas, in patients with double lumen catheters and single lumen catheters it was only 38% and 22% respectively. The incidence of CRBSI was highest in patients with triple lumen catheter as compared in patients with double and single lumen catheters in situ.

### 3.7. Predisposing Risk Factors

#### 3.7.1. Antibiotics Prior to Catheter Insertion

There is no role for empirical antibiotics as the majority of patients (81.5%) developed infections despite being under antibiotic cover.

#### 3.7.2. Duration of Catheter In Situ

Duration of catheter in situ is a predisposing risk factor (*P* = 0.04) for the development of catheter-related infections. The mean duration of catheter in situ (in days) was higher among cases than controls (14.1 versus 10.9).

As shown in Table 4, after logistic regression analysis we found that chance of infections is 2.21 times more among patients with catheters in situ for more than 12 days. Triple lumen catheters imposed a 40-fold increased risk than single lumen catheters for development of catheter-related infections. Patients with femoral venous catheters have an increased risk for catheter-related infections by 20.48-fold when compared to patients with subclavian venous catheters.

## 4. Discussion

Our study investigated the incidence, microbiological profile, clinical profile, and the risk factors for the development of catheter-related infections in a tertiary care hospital. The commonest isolates among the patients with CRLI were Gram-negative organisms (59.0%) while Gram-positive organisms (64.0%) caused the majority of the CRBSI. Overall, in the entire study *S. aureus* was the commonest pathogen isolated accounting to 19.44% followed by CONS (16.67%), *P. aeruginosa* (16.67%), *A. baumannii *(13.88%),* Candida* species (11.1%),* E. coli *(10.18%),* K. pneumoniae *(9.26%), and polymicrobial (2.77%). Overall multidrug resistant organisms accounted for 31.48% of the infections.* Candida* species were isolated from 11.1% of the patients. Although this is lower than available Indian statistics [6, 7], it is still higher than the data published by The National Nosocomial Infections Surveillance System in the United States.

All resistant *Staphylococcus* (MRSA and MRCONS) isolated from CRLI were 100% sensitive to vancomycin, teicoplanin, and linezolid and all sensitive *Staphylococcus* (MSSA, MSCONS) were 100% resistant to ciprofloxacin. All resistant *Staphylococcus* (MRSA) isolated from CRBSI were 100% sensitive to cotrimoxazole and chloramphenicol among routine antibiotics and 100% sensitive to vancomycin, teicoplanin, and linezolid among reserved antibiotics. All sensitive *Staphylococcus* (MSSA and MSCONS) were 100% resistant to ciprofloxacin. The most sensitive routine antibiotic for *P. aeruginosa* strains isolated in CRLI was piperacillin (85.7% sensitive) and the most sensitive reserved antibiotics were cefoperazone-salbactum, piperacillin-tazobactum, ticarcillin-clavulanic acid (85.7% each), and meropenem (78.6%). The most sensitive routine antibiotics for *P. aeruginosa *isolated from CRBSI were piperacillin and ciprofloxacin (100% sensitive each). Among the reserved antibiotics the most sensitive were cefepime, cefoperazone-salbactum, piperacillin-tazobactum, ticarcillin-clavulanic acid, and meropenem (100% sensitive each).

The most sensitive routine antibiotic for *E. coli* isolated in CRLI was cefuroxime (88.9% sensitive) and the most sensitive reserved antibiotic was meropenem (88.9% sensitive). There was no routine antibiotic sensitive for *E. coli* isolated from CRBSI and both were ESBL producers and among reserved antibiotic meropenem was the most sensitive (100%). The most sensitive routine antibiotics for *K. pneumoniae* isolated in CRLI was amikacin (12.5% sensitive) and the most sensitive reserved antibiotic was meropenem (50% sensitive). The most sensitive routine antibiotics for *K. pneumoniae* isolated from CRBSI were gentamicin, netilmicin, amikacin (all 100% sensitive), and meropenem among reserved antibiotics (100% sensitive).

The most sensitive routine antibiotic for *A. baumannii *isolated in CRLI was amikacin (35.7% sensitive) and cefoperazone-salbactum in reserved antibiotic (35.7% sensitive). The only one strain of *A. baumannii* isolated from CRBSI was resistant to all routine and reserved drugs (multidrug resistant).The CRBSI incidence density at our hospital is 8.75 per 1000 catheter days. This is similar to observations from meta-analyses by the National Nosocomial Infections Surveillance System in the United States which reported an incidence density ranging between 2 and 11.3 per 1000 catheter days, as shown in Table 5. Pawar et al. [6] reported a CRBSI incidence density of 4.01 per 1000 catheter days in their study done with one thousand three hundred fourteen participants admitted into a cardiac ICU at New Delhi. Several variables have been quoted as contributing to catheter-related infections. These include the number of catheter lumens, cannulation site, and duration of catheterization. In our study, femoral venous access was associated with a significantly higher incidence of local catheter infections (30.1% versus 26.5% versus 16.9%, resp.) and CRBSI (40% versus 8% versus 36%, resp.) than jugular and subclavian access; jugular access was associated with a significantly higher incidence of local catheter infections than subclavian access (26.5% versus 16.9% resp.). The odds ratio when comparing the incidence of catheter-related infections between femoral versus subclavian venous sites was 20.48 (*P* = 0.000).

In our study the incidence of catheter-related infection was highest with triple lumen catheters when compared with double and single lumen catheters (39.8% versus 37% versus 23.1%). By logistic regression analysis we were able to derive an odds ratio of 35.9 for triple lumen catheters (*P* = 0.002). The duration of catheterization was a significant factor that determined the development of catheter-related infections. Although previous studies have confirmed that central venous catheterization longer than 5 to 7 days was associated with a higher risk of catheter-related infection [8–11], the mean duration of catheterization in our study was 12.32 days and no attempts were made to replace catheters as the CDC guidelines of 1996 [12] and 2002 [1] recommended against routinely replacing CVCs to prevent catheter-related infections. The risk of infection for catheters placed for more than 12 days was 2.21 times when compared with those in situ for less than 12 days (*P* = 0.016).

As shown in Table 6, the number of MRSA isolated in our study is higher than that observed by Pawar et al. (42.8% versus 11.7%) [6]. 19% of enteric bacilli (*E. coli* and *K. pneumoniae)* were ESBL producers. Overall 31.48% were multidrug resistant strains.

There are few limitations on this study. False positive culture may result if skin contacts with catheter. If portion of the catheters not inside the patient is submitted, both false positive and false negative cultures are possible. Potential pathogens may die if the removed catheter is not transported to the laboratory promptly. Different insertion sites were not randomly assigned. No randomized trials, however, have compared infection rates for CVCs placed in the three different sites. Only in the study of Merrer et al. [13] patients were randomly assigned to undergo CVC at the femoral or subclavian site. Not every vascular catheter inserted during the study period was sampled. Patients with local catheter infections were not defined into exit site infections or localized catheter colonization as the numbers were too small. There were few limitations on this study; cases and controls groups are not randomly assigned as all patients were catheterized and during followup, the group that developed local or bloodstream infections were taken as cases, while those that did not develop CRLI became controls. 

## 5. Conclusion

The increasing rate of CRBSI is a matter of concern to our hospital setup. This work will highlight the common microorganisms responsible for CRBSI, their antibiotic susceptibility pattern and also will correlate the clinical significance. This work will help both the clinicians as well as microbiologists in better management of patients as well as in prevention of nosocomial bloodstream infection, especially due to multidrug resistant organisms. 

## Figures and Tables

**Table 1:** Clinical profile of study population.

Clinical profile	Controls (*n* = 50)	Local catheter infections (*n* = 39)	Catheter related blood stream infections (*n* = 11)
Gender			
Male	32 (64%)	26 (66.7%)	8 (72.7%)
Female	18 (36%)	13 (33.7%)	3 (27.3%)
Mean age	43.53	42.10	42.84
Mean hospital stay (in days)	22.71	29.66	28.56
Premorbidities			
Diabetes	4 (8%)	14 (35.9%)	3 (27.3%)
Renal failure	18 (36%)	11 (28.2%)	4 (36.4%)
AIDS	0	0	1 (9.1%)
Malignancies	3 (6%)	1 (2.57%)	0
Indications for catheter insertion			
IV fluids/antibiotics	28 (56%)	30 (77.1%)	7 (63.7%)
Haemodialysis	22 (44%)	11 (28.2%)	4 (36.4%)
Chemotherapy	2 (4%)	1 (2.57%)	0
Local signs of inflammation	0	38 (97.4%)	11 (100%)

**Table 2:** Various catheter characteristics based on lumen size.

Number of lumens	Cases (%) *N* = 50	Controls (%) *N* = 50
Single	11 (22%)	8 (16%)
Double	19 (38%)	29 (58%)
Triple	20 (40%)	13 (26%)

**Table 3:** Site of venous cannulation.

Site of venous cannulation	Cases (%) *N* = 50	Controls (%) *N* = 50
Basilic vein	11 (22%)	7 (14%)
Femoral vein	18 (36%)	8 (16%)
Jugular vein	11 (22%)	21 (42%)
Subclavian vein	10 (20%)	14 (28%)

**Table 4:** Predisposing factors for development of catheter-related infections.

Variable	Odds ratio (95% CI)	“*P*” value
Duration of catheter in situ		
<12 days	1	
>12 days	2.21 (1.16, 4.20)	0.016
No. of catheter lumens		
Single	1	
Triple	35.90 (3.11, 414.26)	0.002
Site of placement		
Subclavian vein	1	
Femoral vein	20.48 (5.71, 73.37)	0.000

**Table 5:** Comparison of various organisms found in different studies.

	Our study, (*n* = 100)	NNIS, 2001 (*n* = 24179)	Pawar et al., 2004 [6] (*n* = 1314)	Subba Rao et al., 2005 [7], (*n* = 135)
Commonest bacteria (in descending order)	*S. aureus* (19.4%) CONS (16.7%) *Pseudomonas* (16.7%)	CONS (31%) *S. aureus* (20%) *Enterococci* (9%)	*E. coli* (47%) *Acinetobacter* (11.7%) *S. aureus* (11.7%) CONS (5.8%)	CONS (32.4%) *Pseudomonas* (31%) *Enterobacter* (13%)

*Candida *	11.1%	9%	11.7%	20%

**Table 6:** Comparison of MDR organisms.

	Our study, (*n* = 100)	NNIS, 2001 (*n* = 24179)	Pawar et al., 2004 [6] (*n* = 1314)
MRSA	42.8%	57%	11.7%
ESBL *Escherichia coli *	27.27%	40%	
